# *GBA* Variants and Parkinson Disease: Mechanisms and Treatments

**DOI:** 10.3390/cells11081261

**Published:** 2022-04-08

**Authors:** Laura Smith, Anthony H. V. Schapira

**Affiliations:** 1 Department of Clinical and Movement Neurosciences, Institute of Neurology, University College London, London NW3 2PF, UK; laura.j.smith@ucl.ac.uk; 2 Aligning Science Across Parkinson’s (ASAP) Collaborative Research Network, Chevy Chase, MD 20815, USA

**Keywords:** Parkinson disease, *GBA*, alpha-synuclein, autophagy, unfolded protein response, lipids

## Abstract

The *GBA* gene encodes for the lysosomal enzyme glucocerebrosidase (GCase), which maintains glycosphingolipid homeostasis. Approximately 5–15% of PD patients have mutations in the *GBA* gene, making it numerically the most important genetic risk factor for Parkinson disease (PD). Clinically, *GBA*-associated PD is identical to sporadic PD, aside from the earlier age at onset (AAO), more frequent cognitive impairment and more rapid progression. Mutations in *GBA* can be associated with loss- and gain-of-function mechanisms. A key hallmark of PD is the presence of intraneuronal proteinaceous inclusions named Lewy bodies, which are made up primarily of alpha-synuclein. Mutations in the *GBA* gene may lead to loss of GCase activity and lysosomal dysfunction, which may impair alpha-synuclein metabolism. Models of GCase deficiency demonstrate dysfunction of the autophagic-lysosomal pathway and subsequent accumulation of alpha-synuclein. This dysfunction can also lead to aberrant lipid metabolism, including the accumulation of glycosphingolipids, glucosylceramide and glucosylsphingosine. Certain mutations cause GCase to be misfolded and retained in the endoplasmic reticulum (ER), activating stress responses including the unfolded protein response (UPR), which may contribute to neurodegeneration. In addition to these mechanisms, a GCase deficiency has also been associated with mitochondrial dysfunction and neuroinflammation, which have been implicated in the pathogenesis of PD. This review discusses the pathways associated with *GBA*-PD and highlights potential treatments which may act to target GCase and prevent neurodegeneration.

## 1. Introduction

Parkinson disease (PD) is the second most common neurodegenerative disorder, affecting over 3% of the population aged over 65 years. The disease is characterised by the progressive loss of dopaminergic neurons in the substantia nigra pars compacta (SNpc) and the presence of intraneuronal proteinaceous inclusions, named Lewy bodies [1]. Towards the end of the 20th century, reports began to emerge associating the lysosomal storage disorder Gaucher disease (GD) with PD [2,3]. GD is an inherited disorder caused by homozygous mutations in the *GBA* gene, which encodes glucocerebrosidase (GCase), a lysosomal hydrolase enzyme which catalyses the catabolism of glucosylceramide (GlcCer) and glucosylsphingosine (GlcSph) [4]. Since then, several large cohort studies have further investigated the link between *GBA* mutations and the risk of developing PD [5,6,7,8]. Approximately 5–15% of PD patients have *GBA* mutations, making them the most important genetic risk factor for PD, occurring more frequently than other genes associated with familial PD including *LRRK2*, *SNCA* and *PARK2* [7].

Over 300 pathogenic *GBA* mutations have been identified [9,10]. These have been associated with loss- and gain-of-function mechanisms. A persistent lack of GCase activity may influence the autophagic-lysosomal pathway (ALP) and has been associated with aggregation of alpha-synuclein. The presence of mutant GCase protein can exert toxic gain-of-function pathways including endoplasmic reticulum (ER) stress and the unfolded protein response (UPR). Dysfunction of mitochondria, the inflammatory pathway and lipid homeostasis have also been implicated in *GBA*-associated PD (*GBA*-PD) and can contribute to the pathogenic accumulation of alpha-synuclein [11].

In this review, we discuss how *GBA* mutations are associated with PD and outline the possible mechanisms involved in the pathogenesis of the disease. Advances in the understanding and identification of the underlying pathways leading to alpha-synuclein accumulation and subsequent neurodegeneration in *GBA*-PD will provide new avenues to be targeted for the development of more efficacious therapies for patients.

## 2. Parkinson Disease

PD is a common neurodegenerative disorder associated with motor and non-motor symptoms. PD patients exhibit a classic triad of motor symptoms including bradykinesia, rigidity and resting tremor. A spectrum of clinically significant non-motor symptoms has also been described. These include cognitive decline, sleep disturbances, hyposmia and psychiatric symptoms [12,13]. It is suggested that at the onset of motor symptoms and PD diagnosis, dopamine neurons in the SNpc are reduced up to 60% [14].

A key feature of PD is the presence of aggregated protein inclusions, Lewy bodies. Lewy bodies are composed of more than 300 proteins, with alpha-synuclein reported to be the most abundant [15,16,17]. Braak et al. proposed a sequential model of Lewy body formation and deposition of alpha-synuclein [18]. This starts at the dorsal motor nucleus of the glossopharyngeal and vagal nerves and anterior olfactory nucleus and then spreads progressively to involve the brain stem and the cortex [18]. The processes by which Lewy body pathology arises and their role in neurodegeneration remain elusive. The leading hypothesis suggests that the pathway of intraneuronal alpha-synuclein aggregation begins with the accumulation of unfolded monomeric species, which can transform into early folded aggregate intermediates and assemble into later-stage β-sheet-rich oligomers, protofibrils and, finally, mature amyloid-like fibrils [19]. It is these fibrils that are the basis of Lewy body formation [20]. Fibrils have long been considered the most toxic alpha-synuclein species, exerting toxicity through several mechanisms including membrane permeability, altered autophagy and mitochondrial dysfunction [21]. However, mounting evidence now indicates that pre-fibrillar forms of alpha-synuclein, such as oligomers, are more critical in the toxicity of alpha-synuclein. Alpha-synuclein oligomers are thought to be the most bioactive and cytotoxic form, causing neuronal dysfunction and death [19,21].

Once aberrant alpha-synuclein accumulates inside a cell, it can either be degraded, deposited in inclusions such as Lewy bodies or released into the extracellular space. These processes may be related to the failure of the cell to properly degrade alpha-synuclein by the ALP [22,23]. In human brains with sporadic PD, there is evidence of autophagic and lysosomal dysfunction [24,25]. This may lead to improper clearance of alpha-synuclein and its subsequent accumulation and aggregation. Another hypothesis arises from evidence of the uptake of extracellular alpha-synuclein fibrils by cells [26,27], which may propagate the spread of alpha-synuclein pathology and act as a template for misfolded, aggregated alpha-synuclein species.

## 3. The *GBA* Gene

The *GBA* gene is located on chromosome 1 (1q21) and is made up of 11 exons. It encodes for the lysosomal hydrolyse enzyme glucocerebrosidase (GCase) (IUBMB enzyme nomenclature number EC 3.2.1.45). The role of GCase is to cleave glycosphingolipids (GSLs) GlcCer and GlcSph into glucose and ceramide, and glucose and sphingosine, respectively. Following its trafficking to the lysosome by the transporter protein LIMP2, GCase catalyses at optimal activity upon interacting with Saposin C, a co-factor, and negatively charged lipids [28,29].

**Figure 1:**
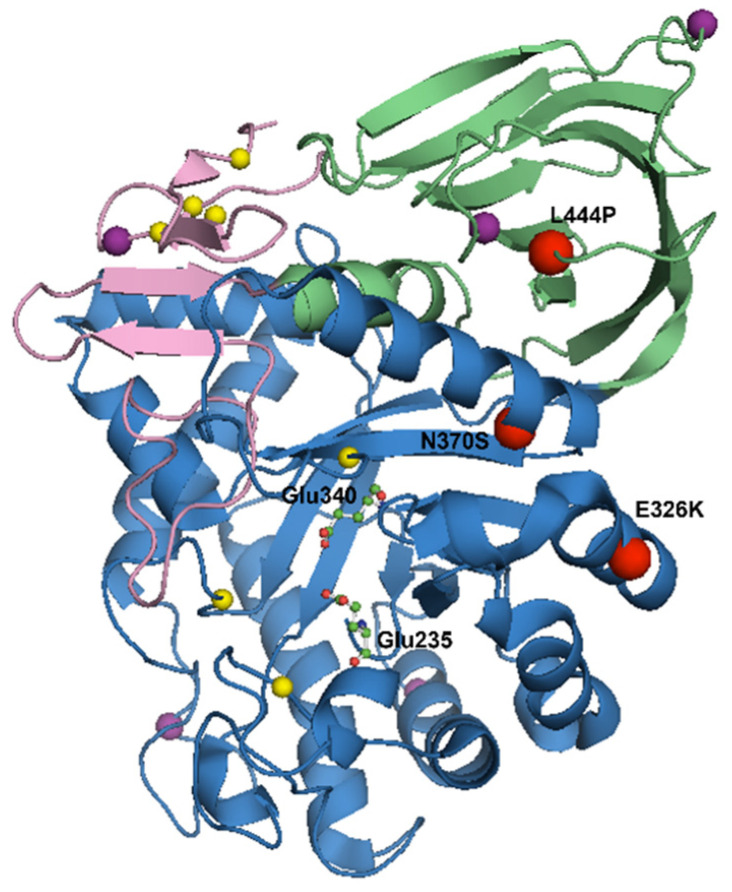
Crystal structure of glucocerebrosidase at pH 5.5 (PDB code 3GXI). Domain I is shown in pink. Domain II is shown in green. Domain III is shown in blue. The active site catalytic residues Glu 235 and Glu 340 are shown as ball-and-stick models. The five *N*-linked glycosylation sites (Asn 19, Asn 59, Asn 146, Asn 270 and Asn 462) are shown as purple spheres. The free cysteine residues are shown as yellow spheres. The three most common *GBA* mutations, L444P, N370S and E326K, are labelled as red spheres. Figure created using The PyMOL Molecular Graphics System, Version 1.2r3pre, Schrödinger, LLC .

The mature GCase protein is composed of 497 residues and is between 59 and 69 kilodaltons depending on post-translational modifications [30]. It is made up of three non-continuous domains: domain I is an antiparallel β-sheet, with two disulphide bridges which may aid proper protein folding; domain II resembles an immunoglobulin fold made up of eight β-sheets; and domain III is composed of a (β/α)_8_ triosephosphate isomerase (TIM) barrel and houses the active site (Figure 1) [31,32].

Homozygous mutations in *GBA* cause Gaucher disease (GD), the most common sphingolipidosis lysosomal storage disorder. GD is a rare, autosomal recessive disease affecting approximately 1 in 800 live births within the Ashkenazi Jewish population [33,34]. Its incidence is lower in the non-Ashkenazi population. Clinically, GD presents as the widespread accumulation of GlcCer and GlcSph within the lysosomes of many cell types, particularly macrophages, across several tissues and organs. It is classified into three subtypes based upon the involvement of the central nervous system (CNS). Type 1 GD is the most common variant and can manifest at any age; this phenotype is normally referred to as non-neuronopathic as it does not usually have any CNS involvement. Types 2 and 3 typically present a more severe clinical phenotype, with disease onset occurring in early life and patients often dying young [35]. These subtypes are often referred to as neuronopathic as they affect the CNS; however, there is a wide spectrum of clinical manifestations across the entire GD subtypes, which suggests that there may be neurological involvement across the whole disease [36]. Neuropathological analysis of type 2 GD patient brains demonstrated neuronal cell loss and astrogliosis, which was absent in type 1 GD patient brains. In the same study, four patients had type 1 GD with parkinsonism, and intraneuronal alpha-synuclein inclusions were observed [37]. Co-cultures of astrocytes and midbrain dopamine neurons from type 1 and type 2 GD patients revealed reduced GCase activity and GlcCer and GlcSph accumulation, which were accompanied by increased alpha-synuclein aggregates when treated with extracellular alpha-synuclein monomers and fibrils, as well as inflammation [38]. These findings suggest a link between alpha-synuclein and *GBA*.

The treatments available for GD are enzyme replacement therapy (ERT) and substrate reduction therapy (SRT). ERT replaces GCase through the administration of recombinant GCase enzymes; these enzymes often have modifications to their terminal mannose residues, allowing for better targeting to and uptake into macrophages. SRT prevents the synthesis of GlcCer and GlcSph, helping to reduce substrate accumulation [39].

## 4. PD and the *GBA* Gene

### 4.1. The Link between GBA Mutations and PD

Interest in the *GBA* gene as a genetic risk factor for PD arose in the 1980s when clinicians noticed a number of type 1 GD patients developed parkinsonism [2,3]. Since then, several large cohort studies have further investigated the link between *GBA* mutations and the risk of developing PD [5,6,7,8]. Further studies have indicated that approximately 5–15% of sporadic PD patients carry a *GBA* mutation, with an overall odds ratio of 5.4 (*n* = 7023) [7,40]. This makes *GBA* mutations numerically the most important genetic risk factor for PD identified to date.

In the normal population, PD occurs in 3–4% of individuals. However, in type 1 GD patients, this prevalence is increased. Interestingly, there does not seem to be a difference between the risk associated with GD patients and heterozygous mutation carriers. It has been estimated that GD patients have a 9.1% chance of developing PD before age 80 years (*n* = 504) [41], although other studies place this as high as 20–30%. Heterozygote *GBA* mutation carriers are just as likely to develop PD before the age of 80, with a US study estimating 7.7% of carriers will develop PD (*n* = 781) [42], while 15% was estimated in a UK cohort (*n* = 220) [43]. In one study of postmortem brains of PD patients, *GBA* mutations were present in 12 of the 57 samples (21%) [44]. These were both homozygous and heterozygous mutations, further confirming that both types are associated with PD.

The frequency of *GBA* mutations varies among different ethnic groups. In the European non-Ashkenazi Jewish population, the frequency is 2.9–12%, whereas in the European Ashkenazi Jewish population, it is 10–31% (*n* = 5691). This is much higher than in the general population, where <1% of healthy individuals are *GBA* mutation carriers [7]. In the Asian population, 1.8–8.7% of people have *GBA* mutations (*n* = 8836), and 2.9–8% of North and South Americans have *GBA* mutations (*n* = 2371) [45,46].

It must be noted, however, that only a minority of GD patients or *GBA* mutation carriers will develop PD. Mutations in the *GBA* gene do not cause a Mendelian form of PD; they are a genetic risk factor and increase the risk of developing PD 5–30-fold, depending on age, ethnicity and mutations included in analysis [7,45,47]. Currently, studies are underway to assess prodromal symptoms of PD in large cohorts of *GBA* mutation carriers to aid in earlier diagnosis and potentially allow researchers to predict who will go on to develop PD [48]. Furthermore, *GBA* mutations have been associated with dementia with Lewy bodies, providing further evidence for a link between *GBA* and alpha-synucleinopathies [40,49].

### 4.2. Presentation of GBA-PD

*GBA*-PD is clinically non-distinguishable from sporadic PD, aside from an earlier age at onset and more cognitive dysfunction [7,47,50,51]. On average, the onset of *GBA*-PD is 5 years earlier than sporadic PD [7,46,52,53].

Much like sporadic PD, *GBA*-PD exhibits the triad of cardinal motor symptoms [54], although progression is more rapid [55,56]. Non-motor symptoms have been reported to be more common and severe in *GBA*-PD compared to non-carriers, with patients often having more advanced clinical decline, with a greater risk for earlier and more prevalent cognitive impairment [7,43,46,50,53,55,56,57,58]. Non-motor symptoms can include reduced cognition, depression, sleep disturbances and anosmia [6,43,59].

The pathology of *GBA*-PD is identical to that of sporadic PD with nigrostriatal dopamine loss and the presence of deposits of aggregated alpha-synuclein in the form of Lewy bodies in the brainstem and cortex [37,46,49,60,61,62]. Some reports suggest that brains from PD patients with *GBA* mutations exhibit a more diffuse pattern of Lewy body distribution throughout the brain, compared to non-carriers [63]; however, other studies demonstrated no difference [64].

Further confirmation of a link between PD and the *GBA* gene arose from a 2020 genome-wide association study. Analysis of the alpha-synuclein gene, *SNCA*, identified a polymorphism that was associated with an increased likelihood of developing PD in *GBA* carriers [65]. The same polymorphism was associated with accelerated motor decline in *GBA*-PD patients, suggesting a role for alpha-synuclein in disease severity [66]. Interestingly, when brain samples from *GBA* mutation carriers who had a diagnosis of PD or Lewy body dementia were analysed, GCase was present in 32–90% of Lewy bodies, compared to non-mutation carriers, where less than 10% of Lewy bodies were GCase-positive [67]. This suggests there may be a direct interaction occurring between GCase and alpha-synuclein.

## 5. Mutations in the *GBA* Gene

To date, approximately 300 pathogenic mutations in the *GBA* gene have been identified [9,10]. These include substitutions, insertions, deletions and complex alleles. The most prevalent mutations are missense mutations, with the point mutations c.1226A > G (N370S) and c.1448T > C (L444P) most commonly associated with GD. Some *GBA* mutations arise from genetic rearrangements and deletions between the functional *GBA* gene and a highly homologous pseudogene (*GBAP*) [7,9,68,69].

The degree of PD pathogenicity associated with each individual *GBA* mutation differs. Some mutations have been stratified into mild or severe. The severity of a *GBA* mutation is based upon the phenotype it presents when homozygous in those with GD. It is thought that mutation severity inversely correlates with GCase activity [70]. Severe mutations are associated with an earlier age of onset and a greater odds ratio for developing PD compared to mild mutations [6,46,47] and may be associated with a higher burden of symptoms, greater cognitive decline and risk of dementia [57,71]. PD odds ratios range between 2.84 and 4.94 for mild mutations and 9.92 and 21.29 for severe *GBA* mutations [6].

The proximity of mutations to the active site is not a reliable predictor of disease severity as disease-causing mutations have been found throughout the entire protein (Figure 1) [72]. For example, the L444P mutation is generally a severe *GBA* mutation although it is located far from the active site. Interestingly, some *GBA* variants, such as E326K, are referred to as risk variants due to the observation that they do not present any clinical features of GD when homozygous, yet increase the risk for developing PD in both homozygous and heterozygous forms [73,74,75]. Along with the severe L444P and mild N370S mutations, the E326K variant is believed to be one of the most prevalent *GBA* variants in PD patients [76,77,78], and patients harbouring this variant have been associated with a severe PD phenotype [79,80,81,82]. This observation suggests that the mechanisms underlying *GBA*-PD may be separate from those leading to GD. A summary of the effects of the N370S, L444P and E326K mutations can be found in Table 1.

## 6. *GBA* Activity and PD

Mutations in the *GBA* gene affect GCase activity differently, with some mutations causing abolition of enzyme activity and others retaining some residual activity [87,88,99,105]. Several mutations occur in and around the active site, which commonly cause GD, and ultimately destabilise the active site to affect GCase activity.

In GD, GCase activity is normally 10–20% of controls, whereas carriers can retain up to 50% [45]. In human brains from *GBA*-PD patients, GCase activity is specifically reduced, with the greatest reduction observed in the SNpc [106]. A reduction in enzyme activity has also been observed in dried blood spots from patients harbouring *GBA* mutations [100], with heterozygotes retaining more activity compared to homozygotes and compound heterozygotes. To date, there is no evidence of a correlation between GCase activity and *GBA*-PD risk.

A link between GCase activity and alpha-synuclein may underlie the relationship between *GBA* mutations and PD. An inverse correlation has been observed between GCase activity and alpha-synuclein accumulation in *GBA*-PD and sporadic PD brains [107,108]. The same has been observed in GCase-deficient mouse, fly and cell models [109,110,111,112,113,114,115,116,117,118]. Recently, midbrain-like organoids deficient in GCase and over-expressing wild-type alpha-synuclein accumulated Lewy body-like pathology, which was absent in organoids with GCase deficiency or *SNCA* triplication alone, suggesting that impaired GCase function promotes alpha-synuclein pathology [119].

Further supporting evidence for a loss-of-function relationship between GCase and alpha-synuclein arises from the observation that enhancing GCase activity can rescue alpha-synuclein pathology [109,120,121]. It has been proposed that there may be a reciprocal relationship between GCase and alpha-synuclein as over-expression of alpha-synuclein results in decreased GCase activity in cell models [122,123]. One study also suggested that pathogenic fibrillar forms of alpha-synuclein may induce a time-dependent reduction in GCase activity in primary neurons and transgenic mice treated with the GCase inhibitor conduritol-b-epoxide (CBE) [124].

Although these studies provide a link between reduced GCase activity and alpha-synuclein pathology, other studies in cell and animal models have failed to demonstrate such a link [109,111,125]. In iPSC-derived dopamine neurons carrying homozygote or heterozygote *GBA* mutations, alpha-synuclein pathology was similar, although GCase activity was significantly lower in homozygotes [92]. Interestingly, in primary neurons and transgenic mouse models treated with CBE, GCase inhibition did not lead to an increase in total alpha-synuclein or the formation of alpha-synuclein pathology but did enhance pre-existing alpha-synuclein pathology, leading to an elevation in pathogenic phosphorylated alpha-synuclein (p-S129-alpha-synuclein) [124]. This finding was not neuron-specific.

Considering that *GBA* mutation carriers are as likely to develop PD as homozygotes even though they retain more activity [43,84,100], and that most GD patients do not develop PD [83] even though GCase activity is very low, it seems likely that loss of activity is not solely responsible for PD onset.

In addition to *GBA*-PD, GCase activity has been reported to be reduced in brains of sporadic PD patients [106,108,126,127,128,129]. A similar reduction has also been observed in the CSF, dried blood spots and monocytes of PD patients with and without *GBA* mutations [100,130,131]. The reports linking a reduction in GCase activity and protein level to sporadic PD confirm the relevance of GCase and its function to the wider PD population.

## 7. Mechanism Underlying *GBA*-PD

**Figure 2:**
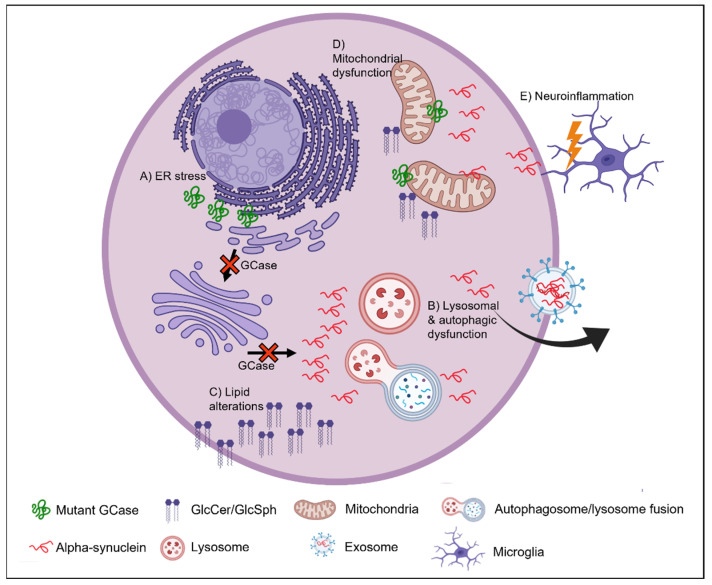
Possible mechanisms underlying the link between GCase, alpha-synuclein and PD. (**A**) *GBA* mutations result in misfolded GCase protein, which is retained in the ER, not trafficked to the lysosome, and activates ER stress pathways such as the UPR. (**B**) Reduced GCase in the lysosome results in lysosomal dysfunction and subsequent impairment of the autophagic-lysosomal pathway. This leads to the accumulation of lipid substrates, GlcCer and GlcSph, and alpha-synuclein. This accumulation can block the trafficking of newly synthesised GCase from the ER/Golgi to the lysosome and further exacerbates lysosomal dysfunction. Impaired degradation of alpha-synuclein through defective lysosomal and autophagic machinery can also lead to an increase in the exosome-mediated release of alpha-synuclein. This mechanism allows alpha-synuclein pathology to propagate through the brain. (**C**) A deficiency in GCase activity at the lysosome can lead to the accumulation of glycosphingolipids, as well as other lipid forms. Aberrant lipid accumulation can affect lipid membrane composition and may enhance the aggregation of alpha-synuclein. (**D**) Defective clearance of mitochondria may occur as a consequence of a GCase deficiency and reduced ALP function. This can lead to the accumulation of defective mitochondria. A GCase deficiency has also been associated with oxidative stress, reduced ATP production and abnormal mitochondrial morphology. (**E**) A GCase deficiency has been linked to neuroinflammation. An accumulation of lipids or alpha-synuclein may activate microglia. Alpha-synuclein released into the extracellular space may also directly bind and active microglia. Created with BioRender.com (accessed on 4 March 2022).

An overview of the possible mechanisms underlying the link between GCase, alpha-synuclein and PD can be found in Figure 2.

## 8. ER Stress

Mutations in the *GBA* gene may lead to the production of a misfolded protein, which can be retained in the ER to induce ER stress [86,91,93]. There is mounting evidence from cell and animal models pointing towards a gain-of-function mechanism for *GBA* mutations that involves ER retention and activation of the pathways associated with ER stress, including ERAD and the UPR [86,90,91,92,132,133]. The extent of ER stress may correlate with disease severity [93,94]. This may be due to more severe conformational changes occurring, affecting protein stability.

In human dopamine neurons and *Drosophila* flies harbouring the L444P and N370S mutations, the activation of ER stress pathways has been demonstrated, and in one study, this was accompanied by increased alpha-synuclein release, providing a link between ER stress and alpha-synuclein homeostasis [86,90,92]. Inhibition of GCase activity can elicit an ER stress response in neuroblastoma cells, indicating that enzyme activity may play a role independent of the presence of a pathogenic mutated protein [134,135]. This suggests that ER stress may occur due to a combination of gain-of-function and loss-of-function mechanisms.

A recent study has suggested that the initial accumulation of alpha-synuclein may cause dysfunction of the ER, leading to the accumulation of misfolded and immature GCase protein [136]. In midbrain neurons from PD patients with *SNCA* triplications, the accumulation of alpha-synuclein led to ER fragmentation and compromised ER protein folding capacity. Immature, misfolded GCase protein was retained in the ER and lacked activity, likely due to the inability of the ER to activate the UPR. This may explain why GCase activity is reduced in sporadic PD and highlights the possibility of both loss-of-function and gain-of-function roles in *GBA*-PD.

The current literature surrounding *GBA* mutations and the ER suggests that early intervention to alleviate ER stress may be an attractive therapeutic avenue to explore to treat *GBA*-PD.

## 9. Autophagic-Lysosomal Pathway

Balance between the synthesis and degradation of molecules and organelles is critical for cellular homeostasis and proper cell function. This is controlled by the ALP which is the cells’ major mechanism of protein clearance and organelle turnover [137]. There are three types of autophagic pathways including macroautophagy, microautophagy and chaperone-mediated autophagy (CMA). There are several key acid hydrolases within the lysosome to help with degradation, including GCase, and when there is defective function, there is impaired clearance [60].

The proper function of the ALP is critical for the degradation of alpha-synuclein [22,23]. Defective ALP has been reported in *GBA*-PD patient brains [106] and neurons [86,92,138]. Several cell and animal models of *GBA* deficiency demonstrate ALP dysfunction [86,118,123,125,137,139,140,141,142]. Impaired autophagic and proteasomal pathways, as a result of GCase deficiency, have also been reported to lead to the accumulation of dysfunctional mitochondria [116,140].

Evidence points toward a correlation between defective ALP and alpha-synuclein pathology in models of GCase deficiency [109,113,115,116,118,123,139]. Impaired ALP mechanisms are evident in *GBA*-deficient neurons and brains [24,86,92,143,144] and accompanied by alpha-synuclein pathology. Furthermore, in cortical neurons from L444P/WT mice, the half-life of alpha-synuclein was increased by more than 70% compared to cells from WT/WT littermates [110], suggesting deficient turnover. A bidirectional loop has been proposed to explain the relationship between *GBA* mutations, alpha-synuclein and the lysosome [123], involving the accumulation of GSLs and alpha-synuclein and prevention of lysosomal trafficking of newly synthesised GCase from the ER which further exacerbates lysosomal dysfunction.

Another link between GCase, alpha-synuclein and CMA has recently been suggested, which involves the mislocalisation of mutant GCase to the surface of lysosomes [145]. In *GBA*-PD human brains, half of the mutant GCase in the lysosome was present on the lysosome surface. This mislocalisation was dependent on a pentapeptide motif in GCase, which is used to target cytosolic proteins for degradation by CMA. Therefore, the binding of mutant GCase to the lysosome prevents CMA, causing the accumulation of CMA substrates including alpha-synuclein. Further analysis in *GBA*-PD dopamine neurons and fibroblasts confirmed defective CMA.

Mounting evidence now points toward the cell-to-cell transmission of alpha-synuclein to propagate pathology around the brain [18]. If alpha-synuclein degradation is impaired, it may be secreted out of the neuron in an exosome-mediated pathway in an attempt to overcome its accumulation [146]. A GCase deficiency has been shown to increase the propagation of alpha-synuclein pathology through the cell-to-cell transmission of toxic alpha-synuclein [147,148], possibly through extracellular vesicle release [149,150]. In N370S dopamine neurons, this increase in alpha-synuclein secretion was coincident with ALP defects [86]. Recently, GCase has been suggested to have a role in the secretion and spread of protein aggregates, as in *GBA*-deficient *Drosophila* flies, protein aggregation was increased through dysregulated extracellular vesicles, and wild-type GCase was able to be packaged and trafficked between cells [151]. In another *Drosophila* study, knock-out of *GBA* resulted in autophagic defects and an abundance of proteins associated with exosome release [152]. Further evidence arises from the analysis of wild-type and L444P/+ mouse brains following a single injection of mouse alpha-synuclein pre-formed fibrils in the striatum. Enhanced propagation of alpha-synuclein pre-formed fibrils was observed in the L444P/+ mouse brain, compared to the control, with widespread alpha-synuclein deposits throughout the brain, suggesting this mutation increases the formation and spread of alpha-synuclein pathology [153].

## 10. Lipid Homeostasis 

Dysfunction of cellular lipid homeostasis may underlie PD pathology. Lipid homeostasis is necessary for synaptic plasticity and neuronal function [154]. The pathological fibrilisation of alpha-synuclein is thought to be strongly mediated by physiological interactions between alpha-synuclein and lipids. A previous study using solution-state nuclear magnetic resonance (NMR) proposed that GCase can directly inhibit lipid-induced aggregation by binding to the C terminal of alpha-synuclein, causing its dissociation from lipids at the N terminal. The same mechanism was also shown to destabilise mature fibrils [155]. However, it remains debatable whether the binding of lipids to alpha-synuclein promotes or prevents aggregation.

Lipid membrane fluidity is essential for the efficient binding of alpha-synuclein [156]. If aberrant lipid homeostasis occurs, this may alter the lipid membrane composition or fluidity and the binding of alpha-synuclein, leading to subsequent neurotoxicity [114,157,158]. Alterations in the lipid composition have been reported in PD brains, including changes in levels of fatty acids and the lipid raft content [159]. Changes in membrane fluidity could greatly affect alpha-synuclein degradation as membrane dynamics are required for macroautophagy and CMA [11].

Alterations in lipid metabolism seem to play a role in *GBA*-PD neurodegeneration. Accumulation of GCase substrates, GlcCer and GlcSph, is a key feature in animal models of *GBA* deficiency [92,111,113,139,157,160,161,162,163]. In a *GBA*-PD mouse model, reducing GSL levels improved cognitive symptoms [160]. Fibroblasts from WT/L444P PD patients have also demonstrated a significant increase in GSLs compared to healthy controls and sporadic PD patients, which correlated with decreased GCase activity [164]. Excess GSLs can alter the lipid membrane composition, leading to changes in membrane fluidity and curvature [165,166].

Currently, the presence of GSL accumulation is yet to be shown in *GBA*-PD brains [167,168]. However, there is evidence of GlcCer and GlcSph accumulation in PD and neuropathic GD brains [126,127,162,169].

A direct link between GSL accumulation and alpha-synuclein fibrilisation has also been suggested. The accumulation of GlcCer has been shown to stabilise toxic alpha-synuclein oligomers and enhance its propagation in cell models of *GBA* deficiency [123,170,171,172]. Lipids extracted from WT/L444P fibroblasts, but not controls, were able to accelerate the aggregation of recombinant alpha-synuclein, due to a higher content of short-chain lipids [164]. A recent study has also demonstrated that over-expression of wild-type GCase in mouse brains reduced the accumulation of lipid-rich alpha-synuclein aggregates, providing further evidence for a role of GCase in lipid and alpha-synuclein homeostasis [173].

A deficiency in GCase has not only been associated with increases in GSL levels, but also with alterations in the composition of other lipid species including ceramide [60,174]. In the SNpc of PD brains [175] and brains from GD patients [176], the marker of lipid-induced stress, glycoprotein NMB (GPNMB), is selectively elevated. This presents further evidence for a primary role for aberrant lipid metabolism in *GBA*-PD degeneration.

Experimental data suggest there may be specific cell types that present a selective vulnerability to lipid alterations. Studies using human brains contain a mixture of neurons and glia, and it may be that substrate accumulation is cell-specific. This highlights the difficulty of determining lipid alterations where small changes may be difficult to detect in certain cell types. Furthermore, it could be that subtle changes in the subcellular localisation of the substrate or alterations in the distribution of species affect alpha-synuclein metabolism.

## 11. Mitochondrial Dysfunction

Mitochondria play a central role in energy production by oxidative phosphorylation. However, they are also heavily involved in other cellular processes including regulation of calcium homeostasis, membrane potential, apoptosis and stress response [177]. Impairment of mitochondrial function is thought to play a key role in PD pathogenesis [178,179,180], and some studies have investigated the link between GCase and mitochondrial dysfunction. In GCase-deficient cells, mice and flies, mitochondrial abnormalities have been observed including oxidative stress, reduced ATP levels, reduced oxygen consumption and abnormal mitochondrial morphology [112,116,140,181]. In *GBA* knock-out flies, these abnormalities were accompanied by an increased sensitivity to oxidative stress, lysosomal dysfunction and impaired autophagic flux.

Although the cause of mitochondrial dysfunction in PD remains unclear, impairment of the ALP may contribute. Defective ALP-mediated clearance of damaged mitochondria has been demonstrated in iPSC-derived dopamine neurons from *GBA* mutation carriers and *Drosophila* fly models [182]. Furthermore, it has also been proposed that the pathogenic accumulation of alpha-synuclein can render dopamine neurons more susceptible to mitochondrial dysfunction induced by 1-methyl-4-phenyl-1,2,3,6-tetrahydropyridine (MPTP) in an L444P/WT mouse model [96], providing a link between GCase, alpha-synuclein and mitochondrial function.

## 12. Neuroinflammation

Neuroinflammation may play an important role in the pathogenesis of PD [183]. High concentrations of inflammatory markers have been observed in the serum of GD patients [184], highlighting a link between *GBA* mutations and inflammation. In animal models of GCase deficiency, there is considerable neuroinflammation including activation of microglia, upregulation of inflammatory cytokines and higher levels of immune markers in the plasma [185,186,187].

It may be that neuroinflammation arises as a result of GlcCer, GlcSph or alpha-synuclein accumulation within neurons, which can activate microglia [188,189,190,191,192]. Alpha-synuclein may be secreted and able to bind directly to Toll-like receptors on the microglia and activate them, resulting in neuroinflammation [193,194]. If an increase in the extracellular release of alpha-synuclein occurs, possibly due to a *GBA* mutation, alpha-synuclein can be taken up into the microglia and astrocytes for degradation [195,196]; however, if these cells are GCase-deficient, then alpha-synuclein degradation may be defective through improper ALP function and thus contribute to the spread of alpha-synuclein pathology.

## 13. GCase as a Therapeutic Target

The link between the *GBA* gene and PD has now opened a new avenue for therapies, with GCase as a novel target. Although dopaminergic therapy and deep brain stimulation (DBS) may be efficacious in alleviating symptoms in *GBA*-PD patients [46,197,198], research is ongoing to develop GCase-targeted therapies to prevent neurodegeneration (Table 2).

Current promising therapies for GD include ERT and SRT. ERT works by administering active, recombinant GCase protein to the cells to increase GCase protein and activity. SRT works to reduce the accumulation of GCase substrates by inhibiting the biosynthesis of GlcCer and GlcSph [199]. Both have shown great efficacy in improving the visceral symptoms of GD but are unable to cross the blood–brain barrier and thus are ineffective in treating neuronopathic symptoms of GD or *GBA*-PD.

Alternative methods to improve the delivery of the recombinant GCase enzyme for ERT are being investigated. These involve ligating a peptide to the GCase enzyme to enhance its ability to cross the blood–brain barrier. The Tat peptide, derived from the transactivator protein of HIV, has been used as Tat-linked cargo proteins have demonstrated increased uptake by micropinocytosis, independent of cell surface receptors [200,201]. Similarly, peptides derived from Rabies virus have been shown to be promising in improving the delivery of brain-targeted proteins [202,203]. When GCase is tagged with such peptides, studies have demonstrated enhanced delivery into neuronal cells compared to untagged GCase, with the ability to reduced lipid accumulation [204]. Preclinical research is also ongoing for the transport-vehicle-modified recombinant GCase enzyme (ETV:*GBA*), which is a transport vehicle platform technology to actively transport enzymes across the blood–brain barrier through receptor-mediated transcytosis [205]. Further studies are required to investigate the efficacy of these methods in treating *GBA*-PD.

Similar to ERT, novel brain-penetrant SRTs are currently under investigation to treat *GBA*-PD. The inhibition of GSL synthesis may reduce alpha-synuclein aggregation and neuronal cell death. Treatment with the SRT miglustat is able to reduce GSL accumulation in dopamine neurons from *GBA*-PD patients, and when coupled with GCase over-expression, this therapy was able to protect against alpha-synuclein toxicity; however, its efficacy is limited as it cannot cross the blood–brain barrier [206]. A potent, brain-penetrant inhibitor of GlcCer synthase, GZ667161 (venglustat), has demonstrated efficacy in reducing alpha-synuclein and GSL accumulation, in addition to ameliorating cognitive dysfunction in a GD synucleinopathy mouse model [160]. Although the initial results from a phase I study of venglustat demonstrated target engagement with no serious adverse effects (ClinicalTrials.gov Identifier: NCT01674036 and NCT01710826) [207], the recent phase II clinical trial showed no benefit and was associated with a decline in motor function in *GBA*-PD (ClinicalTrials.gov Identifier: NCT02906020), suggesting that this drug is ineffective in treating *GBA*-PD. Further development of brain-penetrant SRTs remains a strategy for PD disease modification.

Gene therapy is another method being explored to deliver active, recombinant GCase protein to the brain. Adeno-associated virus (AAV) is a viral vector used for gene delivery into the brain and can deliver to the host cell nucleus without integration into the host genome [208]. AAV-mediated expression of human recombinant GCase in the hippocampus of a pre-symptomatic mouse model of GD has been shown to be effective in reducing alpha-synuclein pathology [111]. Further studies in a symptomatic GD mouse model and in a transgenic mouse model over-expressing alpha-synuclein showed that when virus-encoding human recombinant GCase was injected into the CNS, there was increased GCase expression and activity, which led to a reduction in the levels of GSLs and alpha-synuclein aggregates [120]. The same was exhibited by an AAV-mediated increase in GCase levels in rodent PD models [209]. In a recent study, an injection of viral vectors containing recombinant GCase was sufficient to enhance GCase activity, reduce the alpha-synuclein burden and prevent neuronal death in the SNpc in a synucleinopathy mouse model [210]. Gene therapy targeting the *GBA* gene using an AAV-9 vector (PR001A) for the treatment of *GBA*-PD is currently in phase I clinical trials (ClinicalTrials.gov Identifier: NCT04127578). The compound is also being tested in infants with type 2 GD (ClinicalTrials.gov Identifier: NCT04411654).

**Table 2:** Potential therapies to target GCase to treat *GBA*-PD.

Treatment	Therapeutic Strategy	Drug Name	Phase in Drug Development	Reference
Substrate reduction	Reduce glycosphingolipid accumulation in the CNS	GZ667161 Venglustat Miglustat	Phase II completed for venglustat	[160,206,207]
Small molecule chaperones	Refold mutant GCase in the ER to improve trafficking to the lysosome and increase activity and stability while reducing ER stress	Ambroxol Isofagomine	Phase II completed for ambroxol	[39,90,138,211,212,213,214,215,216,217,218,219,220,221,222,223,224]
Gene therapy	Replace GCase activity and protein levels in the CNS	AAV-mediated delivery of recombinant GCase	Preclinical research ongoing Phase I/II ongoing for PR001 gene therapy (Prevail Therapeutics)	[111,120,205,209,210,225]
GCase activator	Increase GCase activity in the brain	BIA 28-6156/LTI-291	Phase I completed	[205,226]
Transport vehicle modified recombinant GCase	Replace GCase activity and protein levels in the CNS	ETV:*GBA*	Preclinical research ongoing	[205]
Histone deacetylase inhibitors	Replace GCase activity and protein levels	LB-205	Preclinical research ongoing	[227,228]

Other promising GCase-targeted therapies undergoing preclinical and clinical trials include molecular chaperones. Since they have the potential to penetrate the blood–brain barrier effectively, small molecular chaperones of GCase have gained much focus recently for the treatment of PD. These compounds can bind misfolded, mutant GCase in the ER, facilitate the correct folding and increase activity and stability whilst aiding in trafficking to the lysosome [229]. Two types of molecular chaperones exist: inhibitory chaperones, which bind to the active site of the GCase protein, and noninhibitory chaperones, which bind to an alternate site of the GCase protein [229].

A number of inhibitory small molecule chaperone candidates have been identified as potential treatments for *GBA*-PD, including repurposed drugs such as ambroxol and isofagomine [39,221]. In fibroblasts and neurons with *GBA* mutations, such chaperones have demonstrated efficacy in increasing the GCase protein level and activity and aided in the trafficking of mutant GCase to the lysosome [39,90,138,213,218,221,222,230]. Both ambroxol and isofagomine have been shown to successfully reduce ER stress and improve symptoms in *GBA*-mutant *Drosophila* flies [90,217,223]. Challenges arise when using inhibitory compounds, as GCase must out-compete the inhibitors in order to gain access to the enzyme active site at the lysosome. This competition requires the drug dosage to be carefully optimised to ensure the inhibitors act solely as a chaperone to successfully refold and deliver GCase to the lysosome, and not as an inhibitor of GCase [231].

Ambroxol is a pH-dependent inhibitory chaperone of GCase [39] with the ability not only to increase GCase activity, but also to reduce alpha-synuclein pathology [90,138,214,215,216]. Ambroxol exhibits its maximal inhibitory activity at the neutral pH of the ER, and when in the acidic environment of the lysosome, it exhibits nondetectable inhibition [39]. Oral administration of ambroxol has demonstrated increased GCase activity in the brain of mice [216] and non-human primates [215]. Trials investigating the safety and efficacy of ambroxol in humans are now underway. In type 1 GD patients, ambroxol was safely tolerated and exerted a positive effect on GCase (ClinicalTrials.gov Identifier: NCT03950050) [212]. In a recent single-centre, open-label, noncontrolled clinical trial with *GBA*-PD and sporadic PD patients treated with increasing doses of the drug, ambroxol was well tolerated and safe. It was found that ambroxol successfully crosses the blood–brain barrier and enters the CSF where it alters GCase activity and protein levels (ClinicalTrials.gov Identifier: NCT02941822) [211]. This suggests that there is successful target engagement of ambroxol with GCase. The next step is to perform a larger trial and study the efficacy of ambroxol in treating *GBA*-PD and idiopathic PD, and this study is currently planned to begin recruitment in late 2022.

To overcome the challenges associated with inhibitory chaperones, there is significant interest in the development of novel noninhibitory small molecular chaperones for the treatment of PD. Two noninhibitory small molecular modulators of GCase have been identified, namely, NCGC758 and NCGC607 [218,222]. Treatment of iPSC-derived dopaminergic neurons from *GBA*-PD patients with these compounds has demonstrated increased lysosomal trafficking of GCase coupled with reduced GSL and alpha-synuclein accumulation [218,220]. Another noninhibitory small molecule is the GCase activator LTI-291, which has undergone a phase I clinical trial and demonstrated safety and tolerability in participants, with the ability to penetrate the brain (Trialregister.nl ID: NTR7299) [226].

An exciting avenue currently being explored is the use of small molecules to modulate GCase via GCase-independent pathways. One example of this is RTB101, which is an inhibitor of rapamycin complex 1 (TORC1). The role of mTORC1 is to regulate autophagy, and inhibition has been shown to increase autophagy and prevent neuronal cell death in a mouse model of Alzheimer’s disease [232] and improve motor function in parkinsonism rats [233]. GCase can also be manipulated by the modulation of misfolded GCase through small molecules targeting proteins that are involved in the refolding of mutant GCase in times of stress. Such compounds include histone deacetylase inhibitors (HDACis), which lead to aberrant acetylation of chaperones such as heat shock protein (Hsp) 90, preventing Hsp90 binding to GCase and the subsequent ubiquitination and proteasomal degradation of GCase, ultimately leading to increased GCase activity in GD fibroblast lines [227,228]. Additionally, compounds such as arimoclomol can activate Hsp70 to enhance the correct folding and localisation of mutated GCase and have been shown to increase GCase activity in L444P fibroblasts [234]. Future studies may involve identifying GCase-independent pathways that are able to be manipulated to enhance GCase trafficking and activity, in order to slow down disease progression in PD and alpha-synuclein models.

Significant progress has been made in the development of brain-penetrant GCase-targeted therapies; however, it still remains unclear which therapeutic strategy is best suited to treat *GBA*-PD in terms of efficacy, safety and reproducibility. A key challenge is the limited understanding of the precise pathways by which individual *GBA* mutations increase the risk of developing PD, and thus there may be vast differences between the effectiveness of therapeutic strategies between patients.

## 14. Conclusions 

The discovery of the link between *GBA* mutations and PD has provided invaluable insight into the pathogenesis of the disease and novel perspectives for GCase-targeted therapies to prevent neurodegeneration. There is growing evidence highlighting the involvement of pathways including the ALP, lipid metabolism, the ER, mitochondria and neuroinflammation in *GBA*-PD, and there seems to be a reciprocal relationship between GCase and alpha-synuclein. However, much is yet to be understood regarding the molecular basis that underlies the increased risk for PD in *GBA* mutation carriers, why different mutations are associated with differential risks and why gain- or loss-of-function pathways are associated with individual mutations. Further, it is important to understand why some *GBA* mutation carriers develop PD, while some do not. Improving our understanding of how *GBA* mutations influence the predisposition to PD is imperative to facilitate the development of novel and efficacious therapeutics to halt disease progression.

## Figures and Tables

**Table 1:** Summary of the most common *GBA*-PD mutations.

Mutation	Penetrance of Mutation	Location of Mutation	Effect on GCase	GD	*GBA*-PD	References
N370S	0.08–71.8%	Interface of domains II and III	Loss of GCase activity Activation of the UPR Alpha-synuclein pathology	Generally mild, non-neuronopathic GD	Lower disease penetrance and a milder clinical phenotype	[5,6,7,40,47,52,73,80,83,84,85,86,87,88,89,90,91,92,93]
L444P	0.06–18.8%	Domain II	Loss of GCase activity Activation of the UPR Alpha-synuclein pathology	Generally severe, neuronopathic GD	Higher disease penetrance and a worse clinical phenotype	[5,6,7,40,47,52,73,80,83,87,88,89,90,91,92,94,95,96]
E326K	2.8–3.88%	Surface of domain III	Reduces GCase activity to a lesser extent than GD-causing mutations	No clinical manifestation	Worse clinical phenotype	[44,73,74,75,79,80,93,97,98,99,100,101,102,103,104]

## Data Availability

This manuscript is a review paper based on a literature review; thus, no primary data are available.
